# NOX1 inhibition attenuates the development of a pro‐tumorigenic environment in experimental hepatocellular carcinoma

**DOI:** 10.1186/s13046-021-01837-6

**Published:** 2021-01-23

**Authors:** Astrid Vandierendonck, Helena Degroote, Bart Vanderborght, Xavier Verhelst, Anja Geerts, Lindsey Devisscher, Hans Van Vlierberghe

**Affiliations:** 1 Hepatology Research Unit, Department of Internal Medicine and Pediatrics, Faculty of Medicine and Health Sciences, Ghent University, Corneel Heymanslaan 10, Blok B, 2nd floor, 9000 Ghent, Belgium; 2 Gut-Liver Immunopharmacology Unit, Department of Basic and Applied Medical Sciences, Faculty of Medicine and Health Sciences, Ghent University, Corneel Heymanslaan 10, Entrance 36 – Floor 3, 9000 Ghent, Belgium

**Keywords:** Hepatocellular carcinoma, Oxidative stress, NOX, Tumor microenvironment

## Abstract

**Background:**

The poor prognosis of advanced HCC and limited efficacy of current systemic treatments emphasize the need for new or combined targeted therapies. The development of HCC is a multistage process in which liver injury appears in a complex microenvironment associated with oxidative stress. NOX enzymes are the main source of ROS during hepatocarcinogenesis and NOX1 in particular has shown correlation with poor prognosis of HCC patients. This study evaluates the effect of pharmacological NOX1 inhibition on the development and progression of HCC and its effect on the tumor microenvironment.

**Methods:**

The *in vitro* cytotoxic effects of the NOX1 inhibitor GKT771 (Genkyotex) on human Huh7 and Hep3B and murine Hepa1-6 HCC cell lines, the human THP1 monocyte cell line and mouse macrophages were evaluated via MTT, LDH activity and CaspGlo® assays. In order to induce *in vivo* HCC, male SV129 wild-type mice received weekly IP injections of diethylnitrosamine (DEN) (35 mg/kg) for 20–25 weeks. Mice were treated with vehicle or GKT771 (30 mg/kg) via oral gavage, daily or twice daily, in preventive and therapeutic studies. The liver damage was evaluated for inflammation, angiogenesis, fibrosis and HCC development via histology, RT-qPCR, multiplex analyses and ROS levels.

**Results:**

A concentration-dependent reduction in cellular activity of the human HCC cell lines without cytotoxicity was observed. GKT771 treatment reduced LPS-induced pro-inflammatory bone-marrow derived macrophage polarization. DEN injections resulted in 100 % tumor formation and the induction of HCC markers which could be reduced by twice daily dosing of GKT771 at early onset of advanced HCC. DEN-induced HCC resulted in an upregulation of pro-inflammatory, angiogenic and fibrotic markers which was less pronounced in GKT771 treated mice in all treatment regimens. In line, liver fibrosis was induced in HCC mice and this to a lesser extend upon GKT771 treatment.

**Conclusions:**

NOX1 inhibition showed to be safe and well tolerated and was able to attenuate the induction of a pro-inflammatory, angiogenic and pro-fibrotic microenvironment suggesting that this might be a promising adjuvant therapeutic strategy in the treatment of advanced HCC.

## Background

Hepatocellular carcinoma (HCC) is the most common primary liver cancer and represents a major global health problem with increasing incidence and significant cancer-related morbidity and mortality. To date, only a limited number of patients is eligible for curative treatment options, and current therapies for advanced stage HCC have limited efficacy with significant side effects. Consequently, there is an urgent medical need for additional systemic therapeutic options [1].

The development and progression of HCC is a multistage process in which a chronic insult (e.g. alcohol abuse, viral hepatitis, obesity, cholestatic liver injury) induces liver injury characterized by a micro-environment abundant of various types of cellular stress, including endoplasmic reticulum stress, cellular DNA damage, necrosis of damaged hepatocytes, and oxidative stress with reactive oxygen species (ROS) production. In the liver, ROS are generated in response to a wide variety of endogenous and exogenous stimuli [2–6]. ROS species play multiple biological roles and are therefore involved in a large number of physiological phenomena, such as host defense, but also posttranslational protein processing, cellular signalling, regulation of gene expression, and cellular differentiation [7–9]. Their production is strongly regulated to avoid the harmful effects of a redox imbalance. In a healthy liver, antioxidant systems such as superoxide dismutase and catalase efficiently remove excess of ROS to ensure cellular homeostasis. In contrast, during chronic liver disease, increased ROS production, as well as decreased activity of the antioxidant systems, result in oxidative stress [10, 11]. Hepatic carcinogenesis is believed to involve ROS-induced DNA damage and/or mitogenic signalling. Liver injury-mediated ROS production has been shown to contribute to mutagenesis and genomic instability, resulting in the induction of apoptosis and tissue damage. However, these mutations may also have the potential to activate oncogenes and/or inactivate tumor suppressors, thereby initiating oncogenesis. Thus, chronic liver disease-mediated persistent hepatocyte death establishes a microenvironment that favors survival and proliferation of hepatocytes harboring oncogenic mutations. In addition, when activated, liver resident Kupffer cells (KCs) release an ‘oxidative burst’ of superoxide and numerous other products with cytotoxic, pro-inflammatory and growth-promoting activity, and attract circulating immune cells to the liver; all contributing to the establishment of a tumor-promoting microenvironment. ROS also contribute to cancer development and progression, by acting as second messengers in disease-driven intracellular signaling pathways controlling cell proliferation, survival, motility and invasiveness, as well as by controlling the reactivity of stromal components that are fundamental for cancer development and dissemination, inflammation, tissue repair, and *de novo* angiogenesis [11–18].

A number of mechanisms are involved in ROS production. NADPH oxidase (NOX) proteins represent the major non-mitochondrial source of ROS. In addition, there is a growing body of evidence demonstrating that one major effect of inflammation-induced cytokine secretion is the upregulation of ROS-producing NOX isoforms. Seven homologues of the cytochrome subunit of NOX have been described (NOX1-5 and DUOX1-2). NADPH oxidases, with the exception of NOX5, are multimeric complexes, dissociated when inactive, consisting of cytosolic factors (p47phox, NOXO1, p67phox, NOXA1, p40phox, and Rac2) and a redox membrane core. They share the capacity to transport electrons from NADPH to oxygen across the plasma membrane, and to generate superoxide and other downstream ROS. Activation mechanisms, subcellular localization and tissue distribution are highly isoform-dependent [7, 8]. Various isoforms, such as NOX1, NOX2, and NOX4, are distinctively expressed in specific hepatic cell types, including KCs, hepatic stellate cells (HSCs), endothelial cells, hepatocytes, and infiltrating leukocytes. Genetic and pharmacological inhibition of NOX1 has been shown to reduce inflammation and fibrosis in experimental liver disease [5, 6]. The dual NOX1/4 inhibitor GKT137831 attenuated liver fibrosis, ROS production and the expression of several fibrotic, inflammatory and proliferative genes in different mouse models [19–21]. In a phase I clinical trial, GKT137831 was found to be safe and well tolerated [22]. Non-phagocytic NOX1 is constitutively expressed, although its genetic transcription is upregulated by hypoxia, growth factors, growth-related agonists, inflammatory mediators and pathogen-associated molecular pattern molecules [8, 23]. Recent research on human HCC samples showed that high NOX1 levels are correlated with a poor prognosis [24, 25]. The involvement of NOX1 in cell proliferation, tumor growth, cell motility, epithelial-mesenchymal transition (EMT) and matrix metalloproteinase-2 production has been shown in *in vitro* studies [13, 14]. NOX1-/- knockout mice and mice with myeloid NOX1 disruption were reported to develop fewer and smaller tumors following diethylnitrosamine (DEN) injection. DEN-injected wild type (WT) mice that received the NOX-1 inhibitor ML171 also developed fewer and smaller hepatic tumor nodules, compared to their vehicle-treated counterparts [26]. This antitumor effect observed in mice treated with a NOX1-specific inhibitor may open up new avenues for HCC treatment in humans.

In order to further investigate the role of NOX1 and its potential as a therapeutic target in the treatment of HCC, we studied the effect of the NOX1 inhibitor GKT771 (provided by Genkyotex) in a DEN-induced HCC mouse model, with specific focus on the inflammatory tumor microenvironment (TME).

## Materials and Methods

### Nox1 inhibitor - GKT310771

GKT310771 is a potent, selective and specific NOX1 inhibitor (Ki = 65 ± 30 nM) with an affinity similar to diphenyliodonium (DPI; Ki = 70 ± 5 nM) that is an irreversible and unspecific flavoprotein inhibitor (Genkyotex, Saint-Julien-en-Genevois, France). GKT310771 is 65-fold less effective against NOX4 (Ki = 4290 ± 437 nM) and does not inactivate NOX2, NOX3 and NOX5. It is specific for NADPH oxidases over other flavoenzymes and exhibits no ROS scavenging or antioxidant activity according to the absence of affinity for xanthine oxidase and DPPH (Ki > 100 µM). An extensive in vitro off-target pharmacological profiling of GKT310771 against various proteins including ROS-producing and redox-sensitive enzymes, as well as recognized drug targets (GPCRs, kinases, ion channels and others) failed to reveal significant inhibition of any tested candidate when used at 10 µM, thereby demonstrating high specificity of GKT310771 for NOX enzymes.

### HCC cell culture

The human Hep3B and murine Hepa1-6 cell line were purchased from American Type Culture Collection (ATCC, Molsheim, France). The human Huh-7 cell line was provided by dr. Francesca Fornari [27]. All cell culturing agents and materials were purchased from Life Technologies, Ghent, Belgium; unless stated differently. Cell lines were cultured according to the ATCC guidelines, incubated at 37 °C under 5 % CO_2_ and cultured in Dulbecco’s Modified Eagle Medium (DMEM; Hep3B and Hepa1-6) or Roswell Park Memorial Institute-1640 (RPMI-1640; Huh-7), supplemented with 10 % Fetal Bovine Serum (FBS) and 1 % Antibiotic Antimycotic Solution (AA; containing 10’000 units penicillin, 10 mg streptomycin and 25 µg amphotericin B per mL). For subculturing, cells were dissociated using 0.05 % trypsin, stained with trypan blue, and counted using the LUNA-FL™ Dual Fluorescence Cell Counter (Logos Biosystems, Annandale, VA, USA).

### Cytotoxicity assays

To determine the concentration-dependent cytotoxicity of the specific NOX1 inhibitor GKT771 (Genkyotex, Saint-Julien-en-Genevois, France), the cells were seeded at a density of 15 × 10^3^ cells per well in 96-well culture plates, and exposed to various GKT771 concentrations (1.25–100 µM in 1 % dimethylsulfoxide (DMSO)-enriched culture medium), or equal volumes of solvent, DMSO-free medium or positive controls (2 µM staurosporin for MTT and 2 % Triton-X for LDH) for 24–48 h.

The 3-(4,5-dimethylthiazol-2-yl)-2,5-difenyltetrazolium bromide (MTT) assay (Roche Diagnostics, Anderlecht, Belgium) was used according to the manufacturer’s protocol to determine the mitochondrial metabolic activity, a measure for cellular viability. Briefly, cells were incubated with 0.5 mg/ml MTT for 2 h at 37 °C. Mitochondrial dehydrogenases degrade MTT into insoluble formazan crystals and the absorbance of these DMSO-solved crystals was measured at 570 nm against a background control of 630 nm with a spectrophotometer (Multiskan Ascent). The results are shown as percentages of the solvent-treated control group.

The supernatant of the treated cells was used to measure the lactate dehydrogenase (LDH) activity according to the manufacturer’s protocol (Biovision, Milpitas, California, USA). The cytosolic enzyme LDH is released into the culture medium following cytotoxic damage-mediated plasma membrane disruption. LDH was quantified via a coupled enzymatic reaction in which LDH catalyses lactate to pyruvate through NAD^+^ reduction. The released NADH is used by diaphorase to reduce a tetrazolium salt into a red formazan product. The absorbance of the latter was measured at 490 nm against a background control of 630 nm.

The luminescent CaspGlo® 3/7 (Promega, Leiden, The Netherlands) was used to measure caspase-3 and − 7 activities of the different adherent cell cultures. Upon cell lysis, the proluminescent caspase-3/7 substrate, which contains the tetrapeptide sequence DEVD, is cleaved by caspase-3/7. This releases aminoluciferin, which in turn is used by luciferase for the production of light, allowing quantification of the apoptotic state of the cell cultures. All cells were incubated in the dark for 2 h with the CaspGlo® 3/7 reagent prior to detection with a luminometer (Fluostar, BMG Labtech, De Meern, The Netherlands).

### Mouse BMDM and human monocyte cell line THP1

Murine bone marrow-derived macrophage single cell isolates were obtained from mice by flushing the femurs and tibiae with ice-cold phosphate-buffered saline (PBS). After centrifugation (7 min; 1200 rpm; 4˚C), the pellet was resuspended in pre-heated (37 °C) DMEM supplemented with 20 ng/ml murine macrophage colony-stimulating factor (M-CSF, PeproTech, London, UK), 50 µg/ml Gentamycin (from 3 mg/ml stock), 10 % FBS and 1 % AA. Cells were seeded in two 10 cm petri dishes per mouse, with each containing approximately 20 × 10^6^ primary bone marrow-derived macrophages (BMDMs) after 7 days of culturing. Medium was refreshed every other day and cells were collected using enzyme-free dissociation buffer. For the human THP1 monocyte cell line, cells were cultured in suspension in RPMI-1640 supplemented with 10 % FBS. Prior to treatment with GKT771, cells were seeded into well-plates and stimulated with 12-O-tetradecanoylphorbol-l3-acetate (PMA, 10 ng/ml) to allow differentiation into macrophages. Concentration-dependent cytotoxicity of GKT771 with and without lipopolysaccharide (LPS)-stimulation (1 µg/ml; Sigma-Aldrich) on mouse and human macrophages was determined via the MTT and LDH assay, as described above.

### Mice

All animal experiments were reviewed and approved by the Animal Ethics Committee of the Faculty for Medicine and Health Sciences, University Ghent (ECD18/50). Five-week-old male SV129 wild-type mice, purchased from Janvier Labs (Le Genest-Saint-Isle, France), were housed at room temperature and constant humidity in a 12-hour controlled dark/light cycle at the animal facility of the Faculty of Medicine and Health Sciences, Ghent University, Belgium. Mice received standard chow (Pavan Service-Carfil, Oud-Turnhout, Belgium) and water *ad libitum* before and during the experiment. The welfare of all animals was evaluated daily and the mice were weighted weekly, during the entire duration of the experiment.

#### Preparation of vehicle and dose formulation

Vehicle consisted of deionized water containing 1.2w% methylcellulose and 0.1w% Polysorbate 80 (Tween 80), which was stirred overnight at 4 °C to allow complete solubilisation. GKT771 was dissolved in vehicle solution. Both solutions were stored at 4 °C, protected from light and stirred for at least 10–15 minutes before oral administration (dosage of 30 mg/kg).

#### Preventive study

**Fig. 1:**
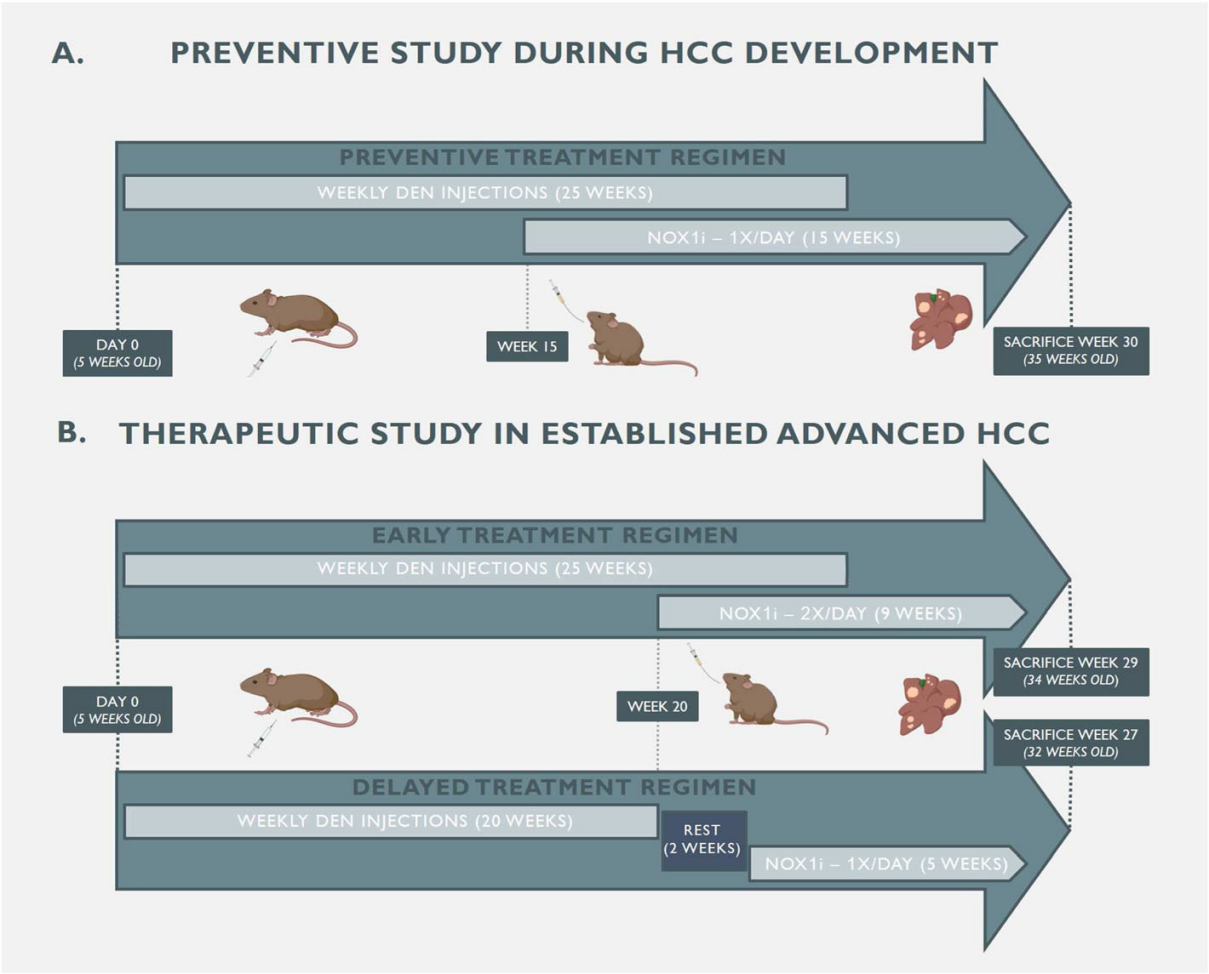
Timeline of in vivo GKT771 studies in DEN-induced HCC. **a** In the preventive study, five-week-old male SV129 wild-type received intraperitoneal injections of saline or 35 mg/kg DEN for 25 weeks to induce HCC. During HCC development (week 15 of DEN), mice were treated daily with vehicle or GKT771 (30 mg/kg) via oral gavage for 15 weeks. **b** In the therapeutic studies, mice received weekly DEN injections for 25 weeks or 20 week in the early and delayed treatment regimen, respectively. In the early treatment regimen, mice were treated with GKT771 twice daily after 20 weeks of DEN for a total of 9 weeks (early euthanasia). Mice in the delayed treatment regimen were kept in a resting period of 2 weeks after the final DEN injection prior to daily GKT771 treatment for 5 weeks

In order to induce HCC, mice received weekly intraperitoneal (IP) injections of saline or diethylnitrosamine (DEN; 35 mg/kg, Sigma-Aldrich, Diegem, Belgium) for 25 weeks. At week 15, mice were randomised into 4 treatment groups and treated for 15 weeks: vehicle-treated healthy saline controls (*n* = 6), GKT771-treated healthy saline controls (*n* = 4), vehicle-treated DEN-induced HCC mice (n = 7) and GKT771-treated DEN-induced HCC mice (n = 10). Mice were treated daily via oral gavage. At week 30 of the experiment, mice were sacrificed (Fig. 1a).

#### Therapeutic study

Two different treatment regimens were applied in the therapeutic setting of the experimental set-up. As in the preventive setting, mice received weekly IP injections of saline or DEN. This was maintained for 25 weeks in the early treatment regimen and 20 weeks in the delayed treatment regimen. Since HCC nodules are macroscopically visible as soon as 20 weeks of DEN [28], an early treatment regimen was started in which mice were planned to be treated with vehicle (*n* = 11) or GKT771 (*n* = 9) twice daily via oral gavage for a total of 10 weeks. However, due to ethical reasons, early euthanasia was performed after 9 weeks. A second treatment regimen was set up in which mice were kept in a resting period of 2 weeks after the final DEN injection at week 20. During this delayed treatment regimen, mice were treated daily with vehicle (*n* = 5) or GKT771 (*n* = 6) for 5 weeks via oral gavage (Fig. 1b).

#### Sample collection

At the day of sacrifice, mice were weighted, and anesthetized by intraperitoneally injecting a ketamine (100 mg/kg) – xylazine (10 mg/kg) solution for blood sampling. The mice were euthanized via cervical dislocation prior to dissection. Both spleen and liver were isolated and weighed, after which the number of nodules was macroscopically evaluated. The lesions of the mice from the preventive study and of the treatment regimen 2 of the therapeutic study were manually separated from the non-tumorous liver tissue. Liver tissue without tumors, the tumors and the total livers were divided into different sections for further analyses. Sections for RNA analysis were collected in RNA-later, snap-frozen using liquid nitrogen and stored at -80 °C until further analysis. Tissue sections for histological examination were collected in cassettes and fixed in a 4 % phosphate-buffered formaldehyde solution, and embedded in paraffin.

### Total RNA extraction

For the quantification of specific gene transcript levels, needle homogenisation was performed on all tissue samples prior to lysis with RLT lysis buffer supplemented with β-mercaptoethanol (Biorad, Temse, Belgium). Total RNA was extracted from all samples using the Aurum™ Total RNA Mini Kit according to the manufacturer’s Spin Format Guidelines (Biorad, Temse, Belgium). The purity and quantity of total RNA was assessed using spectrophotometry (Nanodrop, Thermo Scientific, Wilmington, USA). Purity was determined using the ratio of absorbance at 260 and 280 nm (only ratios between 1.8 and 2.0 were accepted).

### Quantitative real‐time polymerase chain reaction

One microgram of total RNA was converted to single strand cDNA by reverse transcription (Sensifast cDNA synthesis kit, Bioline Reagents Ltd, Kampenhout, Belgium), following the cDNA Bio thermocycler program (10’ 25 °C, 15’ 42 °C, 5’ 85 °C). The cDNA was diluted 1/10 prior to real-time quantification via reverse transcriptase quantitative real-time polymerase chain reaction (RT-qPCR) using SYBR Green (NO-ROX, Sensimix, Bioline Reagents Ltd., Waddinxveen, The Netherlands) according to the manufacturer’s guidelines. All reactions were performed in duplicate. All used primer sequences (Biolegio, Nijmegen, The Nederlands) are listed in Table 1. A real-time cycling program (initial denaturation: 10’ 95 °C; 40 PCR cycles: denaturation 10” 95 °C and annealing/extension/fluorescence reading 60” 60 °C) was run on a Lightcycler® 480 II (Roche, Machelen, Belgium) and melting curve analysis was performed to assess primer specificity. The threshold cycle (Ct) values were further analysed using the 2^−ΔΔCT^ method. In short, the average of the in duplo Ct values of the household and target genes were calculated. The geometric mean of these values of the stable household genes (determined via GeNorm) were calculated and subtracted from the in duplo averages of the target genes (= ΔCp). Next, the mean of the ΔCp of the control samples was subtracted from the ΔCp of each individual sample (= ΔΔCp) and the power was calculated (= 2^−ΔΔCT^). All expression analysis graphs display these powers, and statistical analysis was performed on the normalized log_10_ transformed data of these powers. Statistical analyses were performed as described below.

**Table 1:** Primers used for the mRNA expression analyses conducted on the murine BMDM and liver tissue samples

Marker	Gene ID	Gene name	Forward primer	Reverse primer
Inflammation	CCR2	C-C Motif Chemokine Receptor 2	ATCCACGGCATACTATCAACATC	CAAGGCTCACCATCATCGTAG
	CCL2	C-C Motif Chemokine Ligand 2	TTAAAAACCTGGATCGGAACCAA	GCATTAGCTTCAGATTTACGGGT
	IL6	Interleukin 6	TAGTCCTTCCTACCCCAATTTCC	TTGGTCCTTAGCCACTCCTTC
	IL1β	Interleukin 1 beta	CAACCAACAAGTGATATATTCTCCATG	GATCCACACTCTCCAGCTGCA
	TNFα	Tumor Necrosis Factor alpha	CATCTTCTAAAATTCGAGTGACAA	TGGGAGTAGACAAGGTACAACCC
Inflammasome	iNOS	Inducible Nitric Oxide Synthase	GGCAGCCTGTGAGACCTTTG	GCATTGGAAGTGAAGCGTTC
	NLRP3	Nod-, LRR- and Pyrin domain-containing Protein 3	ATCAACAGGCGAGACCTCTG	GTCCTCCTGGCATACCATAGA
	Caspase 1	Caspase 1	AATACAACCACTCGTACACGTC	AGCTCCAACCCTCGGAGAAA
Immune suppression	PDL1	Programmed death-ligand 1	GACGCAGGCGTTTACTGCT	GCGGTATGGGGCATTGACTTT
HCC	AFP	Alphafetoprotein	AGCTTCCACGTTAGATTCCTCC	ACAAACTGGGTAAAGGTGATGG
	GP3	Glypican 3	TCGACAGCCTCTTTCCCAGTCA	GGTCACGTCTTGCTCCTCG
Stemness	MDR1	Multidrug Resistance 1	AGCCGTAAGAGGCTGAGGCCG	TCACGTGCCACCTCCGGGTT
	EPCAM	Epithelial Cell Adhesion Molecule	GCGGCTCAGAGAGACTGTG	CCAAGCATTTAGACGCCAGTTT
	CD44	Cluster of Differentiation 44	TCGATTTGAATGTAACCTGCCG	CAGTCCGGGAGATACTGTAGC
	CD133	Cluster of Differentiation 133	CTCCCATCAGTGGATAGAGAACT	ATACCCCCTTTTGACGAGGCT
Angiogenesis	CD31	Cluster of Differentiation 31	TGCCTTGTTCATGTTGGGTA	CCTCAGAATATTCCAGGGCA
	END	Endoglin	CCACCGGCCATGAACTTGTCCC	AGTGGGGTGAGGAGATCCCAAGG
	iCAM	Intercellular Adhesion Molecule 1	GCCTTGGTAGAGGTGACTGAG	GACCGGAGCTGAAAAGTTGTA
	vCAM	Vascular Cell Adhesion Protein 1	TGCCGAGCTAAATTACATATTG	CCTTGTGGAGGGATGTACAGA
Fibrosis	αSMA	Alpha-Smooth Muscle Actin	CCAGCACCATGAAGATCAAG	TGGAAGGTAGACAGCGAAGC
	COL1A1	Collagen type 1 alpha	GCTCCTCTTAGGGGCCACT	CCACGTCTCACCATTGGGG
	TGFβ	Transforming Growth Factor beta	TGAGCGTCACTGGAGTTGTACGG	GGTTCATGTCATGGATGGTGC

### Luminex

Snap-frozen liver tissue was defrosted in 1 mg/mL protease inhibitor cocktail (cOmplete™, Mini, EDTA-free Protease Inhibitor Cocktail, 04693 159 001, Roche, Sigma-Aldrich, Overijse, Belgium), 1 v% phosphatase inhibitor cocktail 2 (P5726, Sigma-Aldrich, Overijse, Belgium) and 1 v% phosphatase inhibitor cocktail 3 (P0044, Sigma-Aldrich, Overijse, Belgium) in PBS, lysed by sonication and centrifuged for 15 min by 15,000 rpm at 4 °C. Supernatant was stored at − 80 °C until further analysis. Total protein concentrations were measured by BCA protein assay following manufacturer’s guidelines (DC protein assay, 500 − 0116, Bio-Rad, Temse, Belgium). Protein levels of TNF-α, MCP-1/CCL2 and IL6 were determined by using Bio-plex Pro Reagent Kit and Luminex microbeads (Biorad, Temse, Belgium) according to the manufacturer’s guidelines.

### ROS assay

Hepatic tissue lysates were prepared by defrosting snap-frozen liver tissue in PBS prior to sonication on ice. The lysates were centrifuged for 5 min by 10,000 g at 4 °C. The supernatant was used for the quantification of ROS levels, which was performed using a commercial DCF ROS/RNS Assay Kit (Ab238535, Abcam, Cambridge, United Kingdom,) according to the manufacturer’s instructions.

### Histological analysis

After deparaffinization and rehydration of the tissue samples, sections of 5 µm of paraffin-embedded liver tissue were stained with Sirius Red (Sigma-Aldrich) for histopathological examination according to the Metavir​ scoring system. This system allows to assess the extent of fibrosis in our liver samples, which was visualised using the Cell^D software (Olympus).

### Statistical analysis

Statistical analyses were performed using GraphPad Prism 8 (GraphPad Software, California, USA). Normality was tested using the D’Agostino & Pearson omnibus test. Outliers were identified with the ROUT method and were removed for further analysis. The maximum desired false discovery rate was set to 1 %. Gaussian distributed data of two groups were compared via t-tests and multiple groups were compared by one-way analysis of variance (ANOVA) test and corrected with the Holm-Sidak test. Non-normally distributed data were either transformed to Gaussian distribution via LOG_10_-transformations or analysed via Kruskal Wallis tests for multiple comparison. Data are presented as the fold change relative to expression in controls as mean ± SD, unless stated differently in the figure legends. P-values are reported two-sided and considered significant when less than 0.05 (*p < 0.05, ** *p* < 0.01, *** *p* < 0.001, **** *p* < 0.0001).

## Results

### Moderate doses of GKT771 treatment do not induce HCC cell cytotoxicity in vitro

**Fig. 2:**
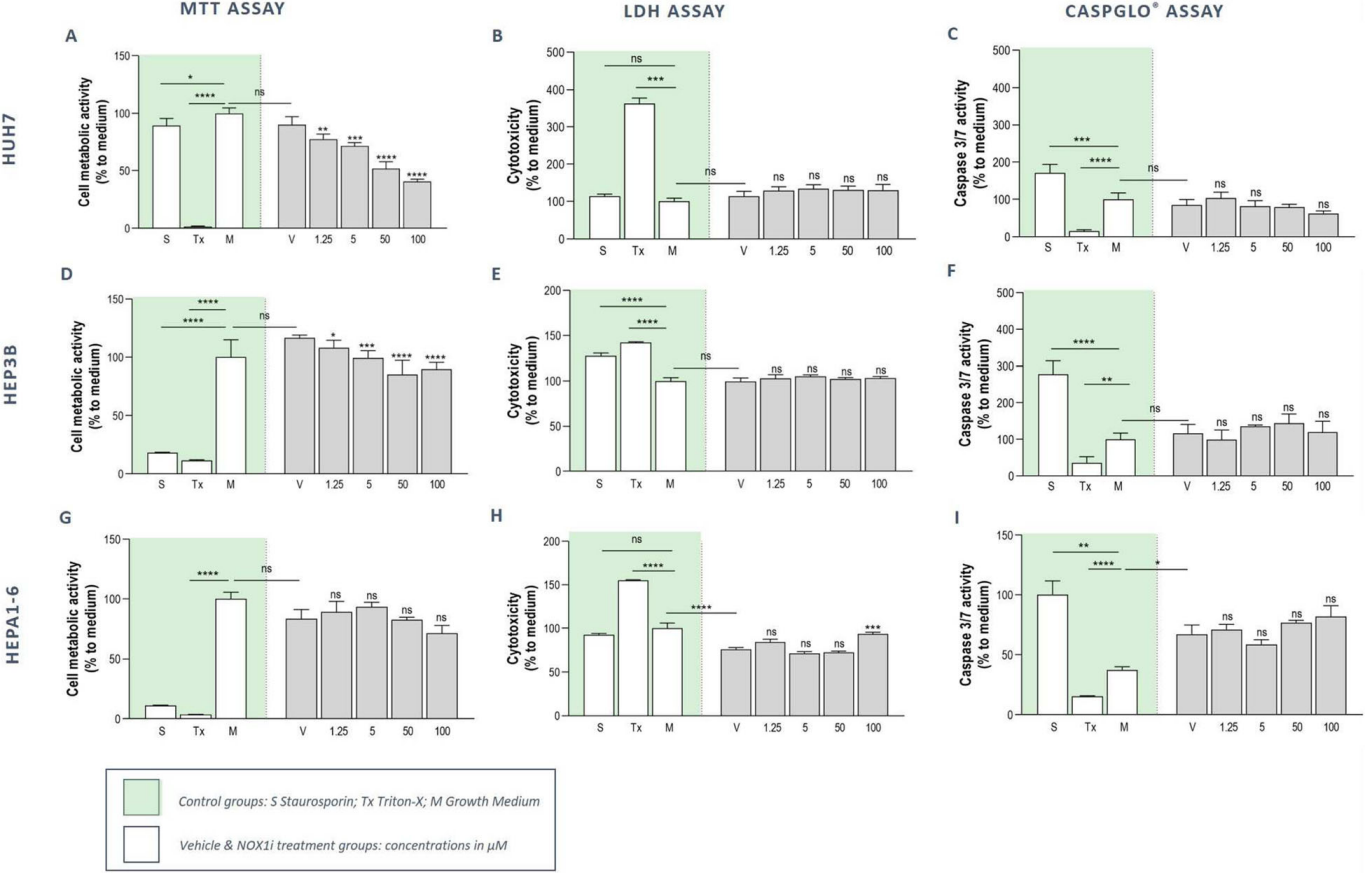
Moderate doses of GKT771 treatment do not induce HCC cell cytotoxicity in vitro. To determine the concentration-dependent cytotoxicity of specific NOX1 inhibition, human Huh7 **a-c** and Hep3B **d-f** and murine Hepa1-6 **g-i** HCC cells were treated for 24 hours with 1.25–100 µM GKT771 (NOXi), vehicle (V), vehicle-free medium (M) or positive controls (S and Tx). MTT (mitochondrial metabolism), LDH (membrane integrity, necrosis) and CaspGlo® (apoptosis) assays were performed. The results are shown as percentages of the vehicle-treated control group. S: Staurosporin; Tx: Triton-X; M: Vehicle-free medium; V: Vehicle. Data shown as mean ± SD, ^ns^not significant; ^*^p < 0,05; ^**^p < 0,01; ^***^p < 0,001; ^****^p < 0,0001 compared to vehicle, unless indicated differently

To assess the potential cytotoxic effects of NOX1 inhibition *in vitro*, two human HCC cell lines (Huh7 and Hep3B) were treated for 24 hours with various concentrations of GKT771, ranging from 0 to 100 µM. Mitochondrial metabolism was examined as an indicator of cell viability using the MTT assay, and was found to be significantly reduced in a concentration dependent manner in both cell lines (Fig. 2a & d). No loss of membrane integrity or increase in caspase 3/7 activity could be detected using an LDH and CaspGlo® assay respectively, which indicates the absence of cell lysis or apoptotic cell death of human HCC cells upon treatment with GKT771 at concentrations up to 100 µM (Fig. 2b-c & e-f).

Identical analyses following a similar GKT771 treatment regimen in the murine Hepa1-6 HCC cell line revealed no significant alterations in mitochondrial metabolism and caspase 3/7 activity (Fig. 2g & i). However, an impaired cellular membrane integrity could be observed at 100 µM, indicating the presence of a moderate toxic effect at the highest concentration (Fig. 2h).

### NOX1 inhibition attenuates LPS-induced macrophage polarization

**Fig. 3:**
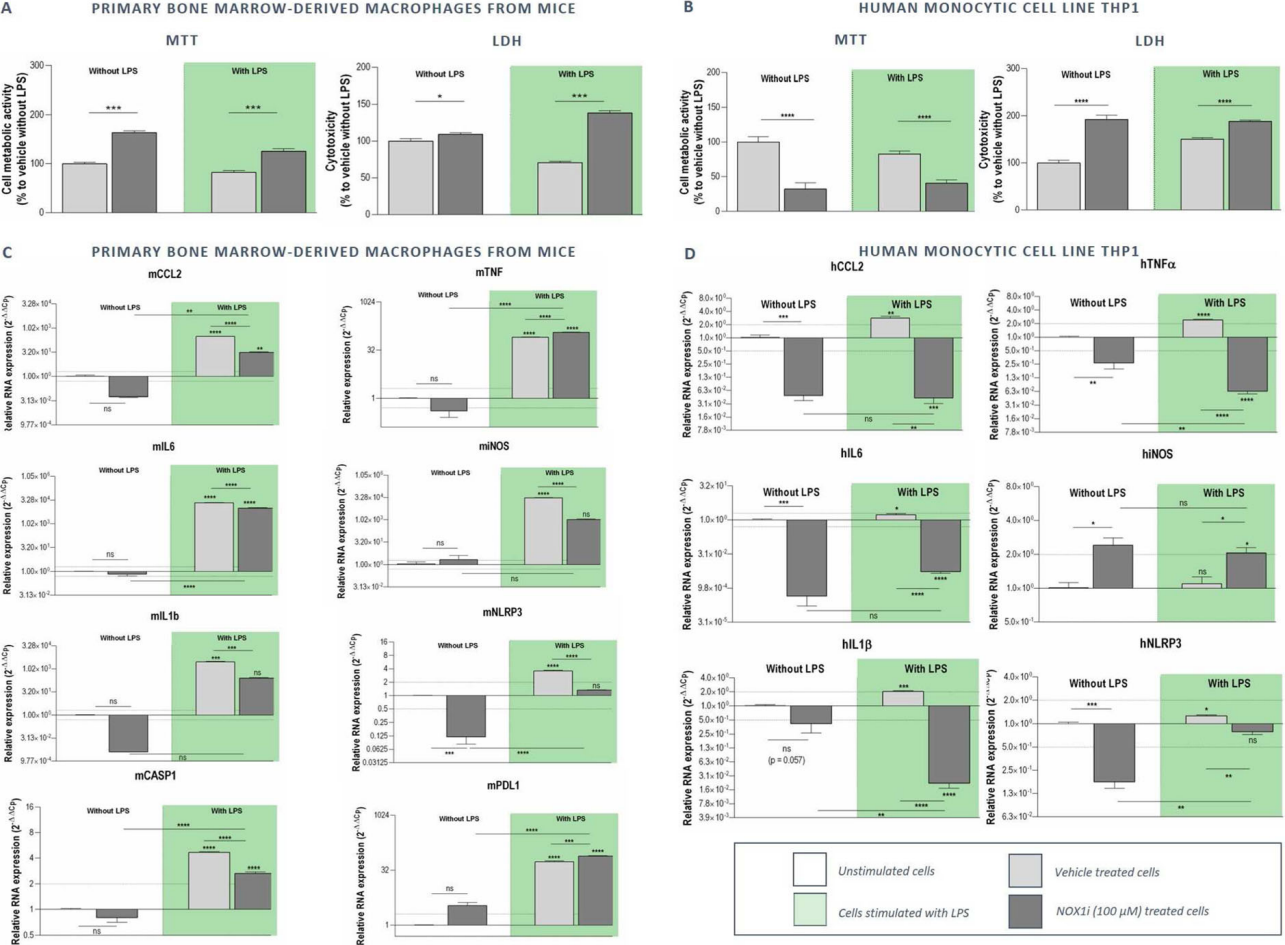
Cytotoxic effects of NOX1 inhibition on mouse BMDMs and human monocyte cell line THP1**.** Murine bone marrow-derived single cell isolates and THP1 cells were cultured in medium containing M-CSF or PMA respectively, to induce differentiation into macrophages. The resulting BMDMs and THP1 cells were treated with vehicle/100 µM of GKT771 for 48 h with and without LPS-stimulation (1 µg/ml). **a-b** MTT and LDH analyses were performed to determine the mitochondrial metabolic state and the disruption of cellular membrane integrity respectively. NOX1 inhibition attenuates LPS-induced BMDM polarization. **c-d** mRNA expression of different inflammatory markers was examined via RT-qPCR. Data presented as relative expression to vehicle-treated controls in the absence of LPS. ^ns^not significant; * *p* < 0,05; ** *p* < 0,01; *** *p* < 0,001; **** *p* < 0,0001 compared to vehicle without LPS, unless indicated differently

Murine bone marrow-derived single cell isolates and THP1 cells were seeded in medium containing M-CSF or PMA respectively, to induce proliferation and differentiation into macrophages. The resulting primary BMDMs and THP1 macrophages were treated with 100 µM of GKT771 with and without LPS-stimulation. MTT and LDH analyses showed a significant enhancement of the mitochondrial metabolic state associated with a disruption of cellular membrane integrity of GKT771-treated BMDMs in both the presence and absence of LPS (Fig. 3a). In the human monocyte cell line THP1, GKT771 induced a decrease in cell metabolic activity and increased cytotoxicity, in both the presence and absence of LPS (Fig. 3b).

To assess the effect of NOX1 inhibition on the polarization of BMDMs *in vitro*, the mRNA expression of inflammatory markers (CCL2, IL6, IL1β, TNFα, iNOS, NLRP3 and Caspase 1) and of the immune suppressive marker PDL1 was examined via RT-qPCR upon treatment of these cells with 100 µM of GKT771, with and without LPS. LPS activation of the murine BMDMs resulted in a significant increased expression of the analysed markers which was significantly less pronounced in combination with NOX1 inhibition for CCL2, IL6, IL1β, iNOS, NLRP3 and Caspase 1 (Fig. 3c). Our results were confirmed in the human monocyte cell line THP1 for the inflammatory markers CCL2, IL6, IL1β and TNFα (Fig. 3d). This shows that NOX1 inhibition is able to attenuate LPS-induced polarization in both murine BMDM and the human monocyte cell line THP1.

### NOX1 inhibition attenuates the development of a pro‐tumorigenic microenvironment during HCC development

**Fig. 4:**
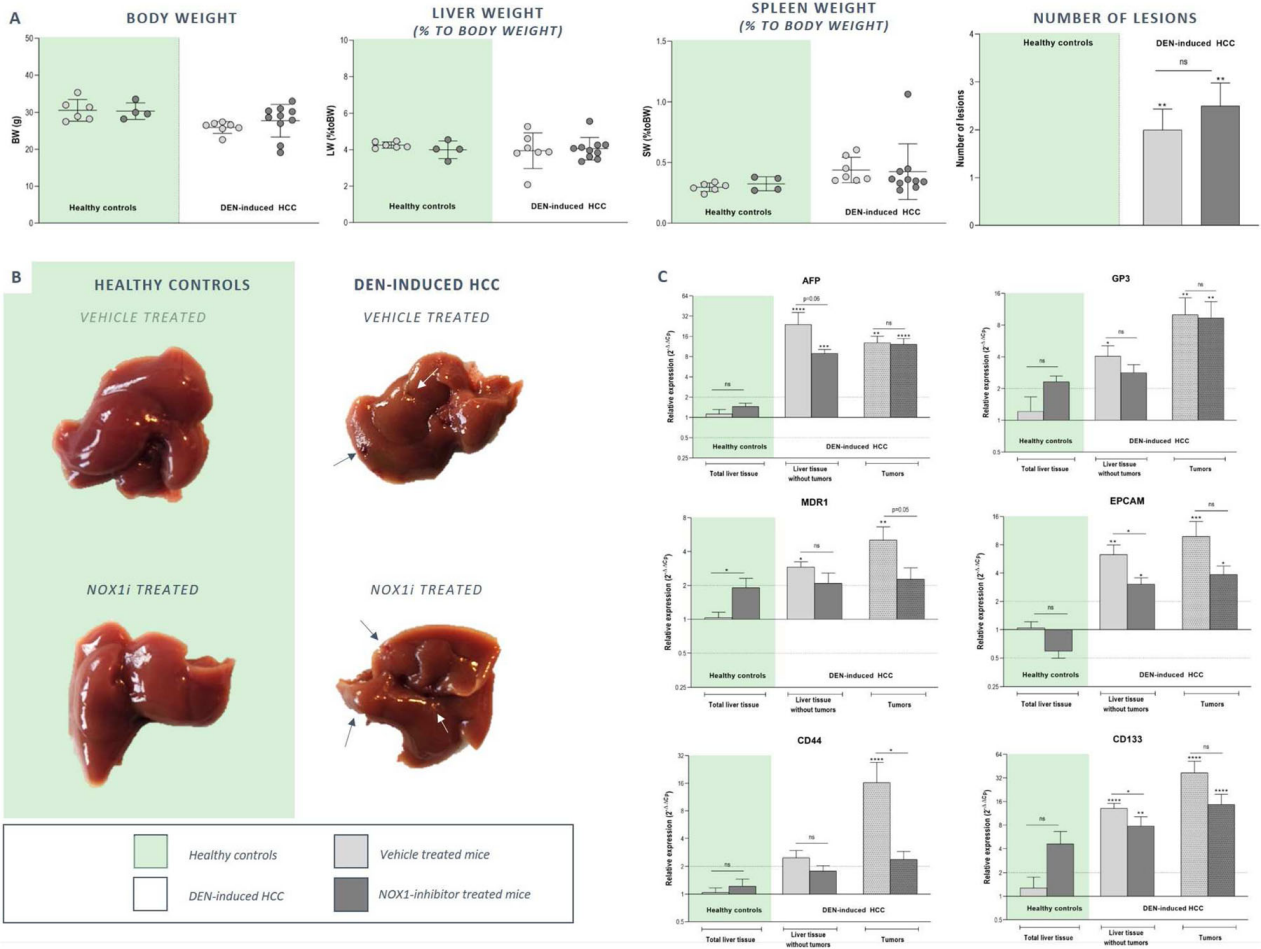
NOX1 inhibition does not prevent HCC development in mice. In order to induce HCC, mice received weekly DEN (35 mg/kg) injections for 25 weeks. At week 15, mice were treated daily with vehicle or 30 mg/kg of GKT771 for 15 weeks via oral gavage. At week 30 of the experiment, mice were sacrificed. **a** Body, liver and spleen weight were recorded and **b** macroscopically visible tumors were counted. NOX1 inhibition attenuates the Induction of hepatic cancer stem cell markers in DEN-induced HCC. **c** The mRNA expression of HCC (AFP and GP3) and cancer stem cell (MDR1, EPCAM, CD44, CD133) markers in the separated tumoral lesions and non-tumoral surrounding liver tissue of both vehicle and GKT771 treated HCC mice was evaluated by RT-qPCR. ^ns^not significant; * *p* < 0,05; ** *p* < 0,01; *** *p* < 0,001; **** *p* < 0,0001 compared to vehicle-treated healthy controls, unless indicated differently

Mice received weekly IP injections of DEN for 25 weeks. At week 15, daily treatment with GKT771 was initiated to evaluate the effect of NOX1 inhibition on tumor development. DEN administration resulted in a mild decrease in body weight and a moderately elevated spleen weight (not significant). As expected, DEN administration induced macroscopically visible tumoral lesions in the liver of both vehicle and GKT771 treated mice, while in the healthy control groups (saline) no lesions were detected. Preventive GKT771 treatment did not significantly alter the total number of tumor lesions in the liver (Fig. 4a-b). In line, the expression of two HCC markers, AFP and GP3, was induced in tumor and surrounding liver tissue of both vehicle and GKT771 treated HCC mice (Fig. 4c). However, significant induction of hepatic cancer stem cell markers (MDR1, EPCAM, CD44 and CD133) in the tumors and surrounding liver tissue was attenuated upon GKT771 treatment (Fig. 4c).

**Fig. 5:**
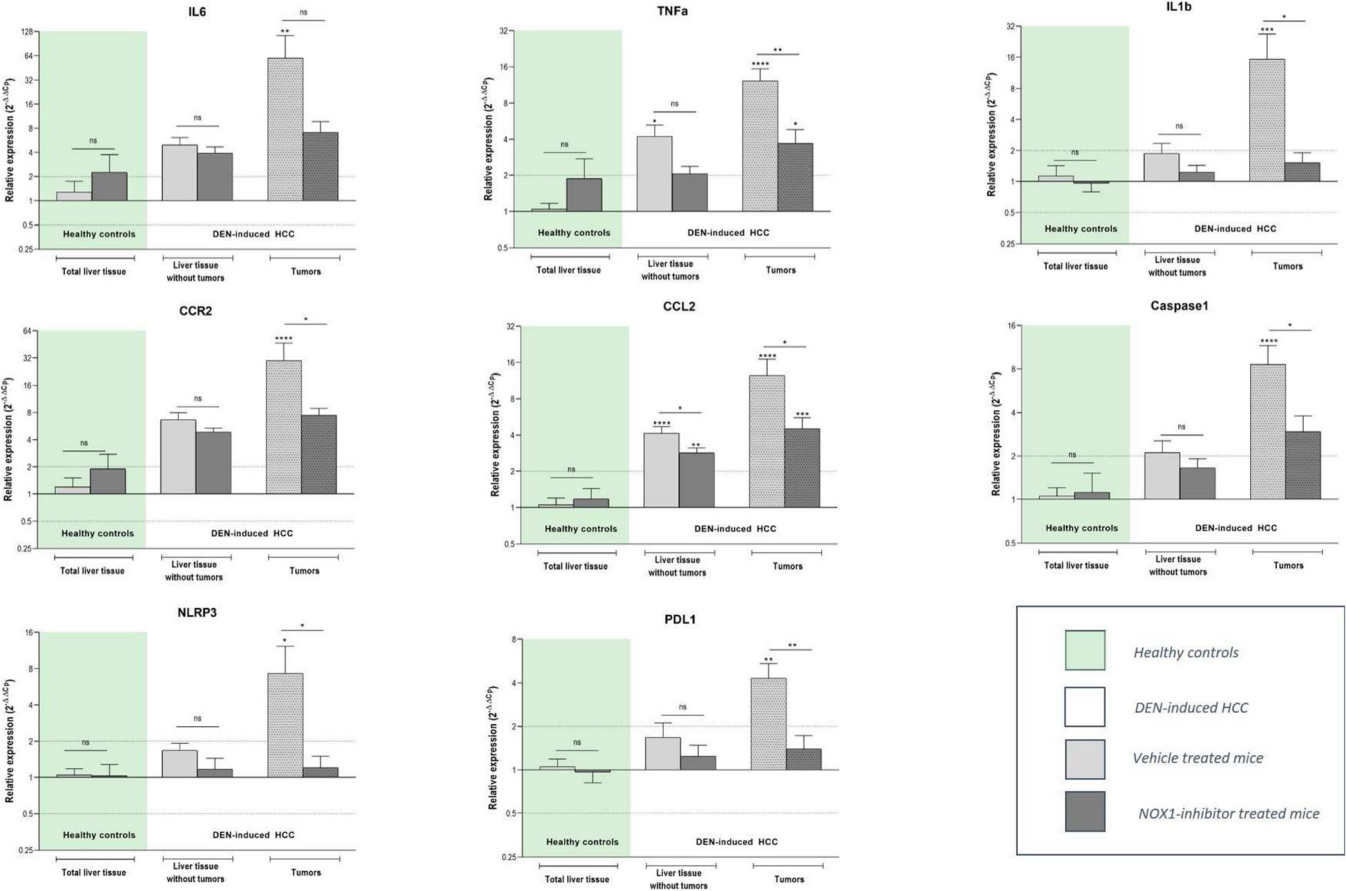
NOX1 inhibition dampens the upregulation of pro-inflammatory markers during HCC development. After 25 weeks of DEN administration and 15 weeks of vehicle/GKT771 treatment, HCC lesions and non-tumoral surrounding liver tissue were sampled for mRNA expression analysis. The expression of inflammatory markers (IL6, TNFα, IL1β, CCR2, CCL2, Caspase 1 and NLRP3) and of the immune suppressive marker PDL1 was performed via RT-qPCR. Data shown as mean ± SEM. ^ns^not significant; * *p* < 0,05; ** *p* < 0,01; *** *p* < 0,001; **** *p* < 0,0001 compared to vehicle-treated healthy controls, unless indicated differently

DEN-induced HCC resulted in an induced expression of inflammatory markers IL6, TNFα, IL1β, CCR2, CCL2, Caspase 1 and NLRP3, and of the immune suppressive marker PDL1, compared to healthy control liver tissue (Fig. 5). This induction was most explicit and significant in the tumor lesions, but also present in surrounding liver tissue. Preventive administration of GKT771 resulted in a less pronounced induction of these markers, both in tumor lesions and surrounding tissue (Fig. 5).

**Fig. 6:**
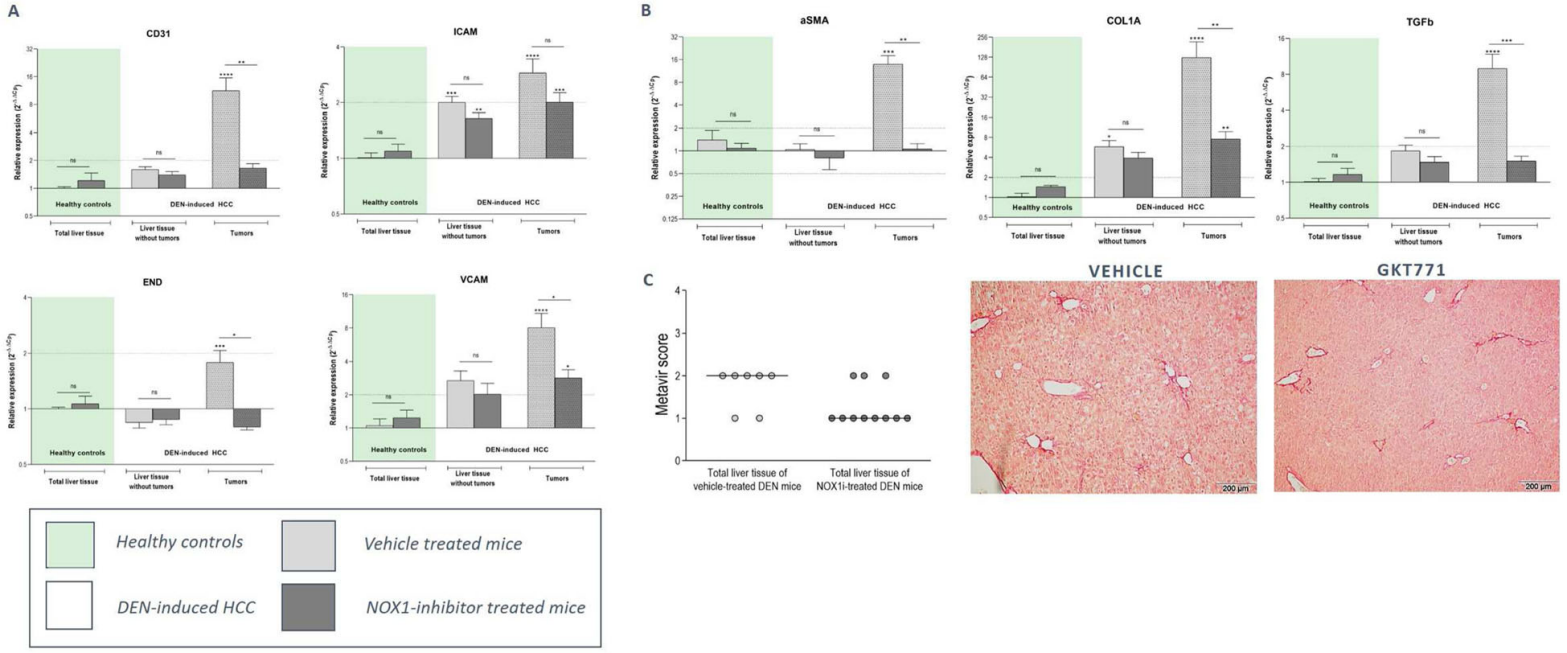
NOX1 inhibition attenuates the induction of angiogenic and fibrotic markers during HCC development. After 25 weeks of DEN administration and 15 weeks of vehicle/GKT771 treatment, HCC lesions and non-tumoral surrounding liver tissue were sampled for mRNA expression analysis. RT-qPCR was performed to determine the expression of **a** different angiogenic markers (CD31, END, iCAM and vCAM) and **b** fibrotic markers (αSMA, COL1A and TGFβ). Data shown as mean ± SEM. ^ns^not significant; * *p* < 0,05; ** *p* < 0,01; *** *p* < 0,001; ****p < 0,0001 compared to vehicle-treated healthy controls, unless indicated differently. **c** Sirius red staining of the livers of DEN-induced HCC mice receiving preventive GTK771 treatment was performed and Metavir scoring was compared to vehicle treated HCC mice. Data shown as individual scoring points with median. Metavir score: F0 - no fibrosis; F1 - fibrosis exists with expansion of portal zones; F2 - : fibrosis exists with expansion of most portal zones, and occasional bridging

To further evaluate the effect of GKT771 on the progressing tumoral microenvironment, mRNA expression analysis of angiogenic and fibrotic markers was performed. NOX1 inhibition was able to attenuate DEN-induced induction of both angiogenic (CD31, END, iCAM, vCAM) and fibrotic (αSMA, COL1A, TGFβ) markers (Fig. 6a-b). Histological analysis of liver fibrosis confirmed this anti-fibrotic effect of NOX1 inhibition early in DEN-induced HCC development (Fig. 6c).

### Early treatment and twice daily dosing of the NOX1 inhibitor is able to attenuate the progression of advanced HCC

**Fig. 7:**
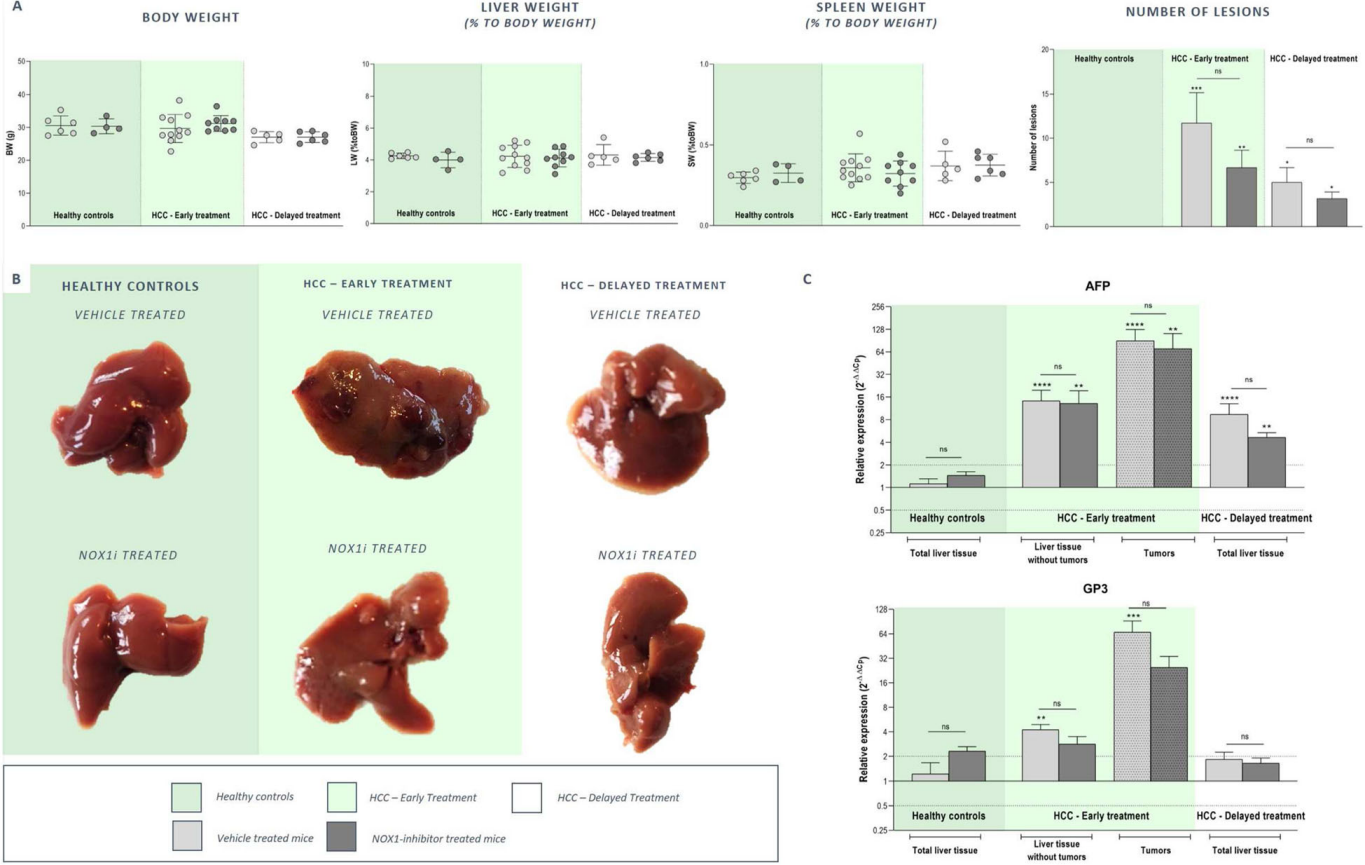
Twice daily dosing of GKT771 at onset of advanced HCC reduces HCC lesions. Mice received weekly DEN (35 mg/kg) injections for 25 or 20 weeks and received twice or single daily doses of vehicle/GKT771 via oral gavage in an early or delayed treatment regimen, respectively. **a** Mice were sacrificed and morphometric data (body, liver and spleen weight) was recorded. Data shown as mean ± SD. **b** Macroscopically visible tumors were counted and subdivided into three categories (large, intermediate, small). Data shown as mean ± SD. ^ns^not significant; * *p* < 0,05; ** *p* < 0,01; *** *p* < 0,001; **** *p* < 0,0001 compared to vehicle-treated healthy controls, unless indicated differently. **c**: mRNA expression of HCC markers (AFP and GP3) in tumors and non-tumoral surrounding liver tissue of vehicle (light grey bars) and GKT771 treated (dark grey bars) mice was determined via RT-qPCR. Data shown as mean ± SEM. ^ns^not significant; * *p* < 0,05; ** *p* < 0,01; *** *p* < 0,001; **** *p* < 0,0001 compared to vehicle-treated healthy controls, unless indicated differently

**Fig. 8:**
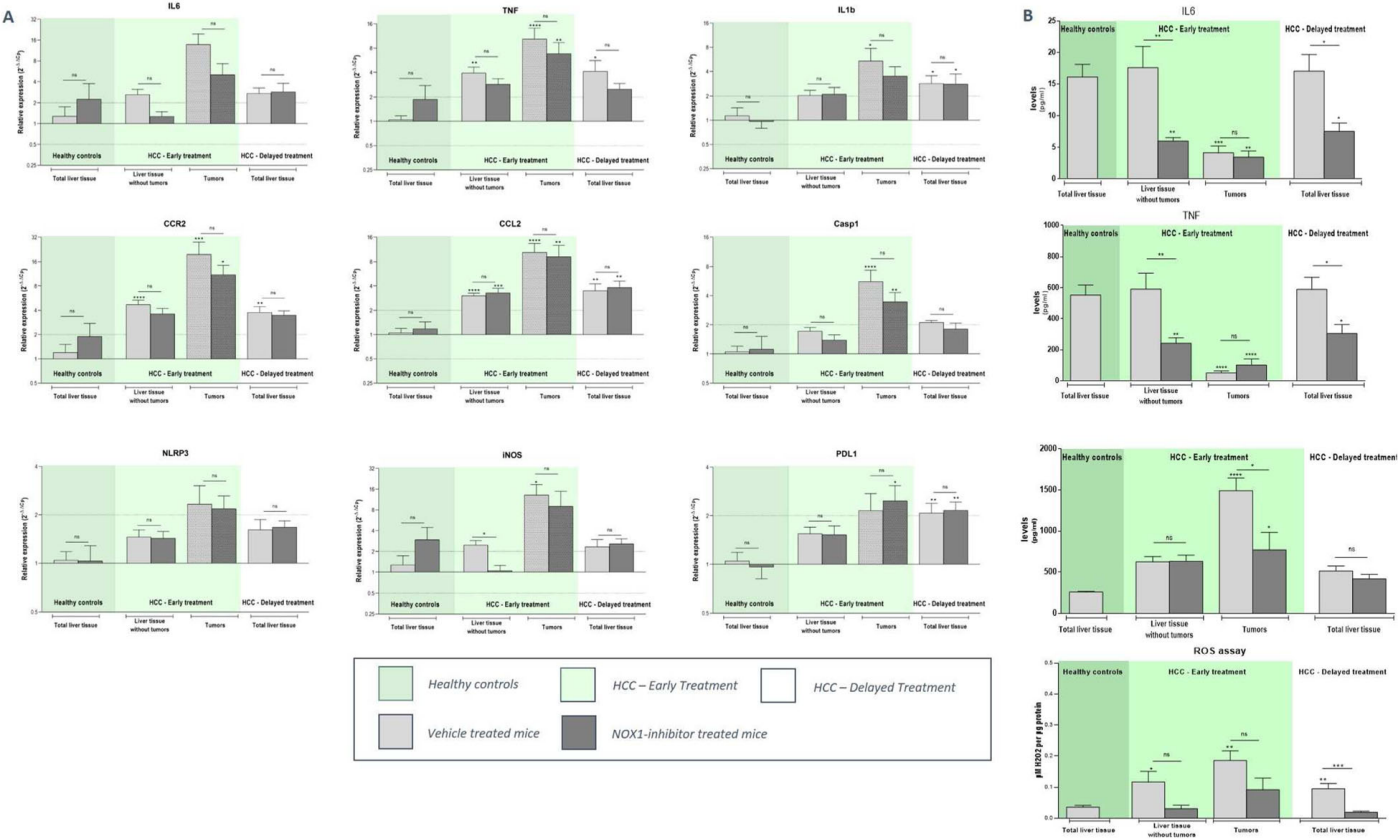
NOX1 inhibition attenuates the induction of the pro-inflammatory microenvironment in established HCC in mice. Mice received weekly DEN (35 mg/kg) injections for 25 or 20 weeks and were administered twice or single daily doses of vehicle/GKT771 via oral gavage in an early or delayed treatment regimen, respectively. Healthy livers, HCC lesions and surrounding tissue was assessed (**a**) RT-qPCR analysis to determine the mRNA expression levels of inflammatory (IL6, TNFα, CCL2, CCR2) and inflammasome (NLRP3, caspase 1, IL1β, iNOS) markers and the immune suppressive marker PDL1. **b** Multiplex analysis of IL6, TNFα, CCL2 levels and ROS levels (µM H202 per µg protein). ^ns^not significant; * *p* < 0,05; ** *p* < 0,01; *** *p* < 0,001; **** *p* < 0,0001 compared to vehicle-treated healthy controls, unless indicated differently

**Fig. 9:**
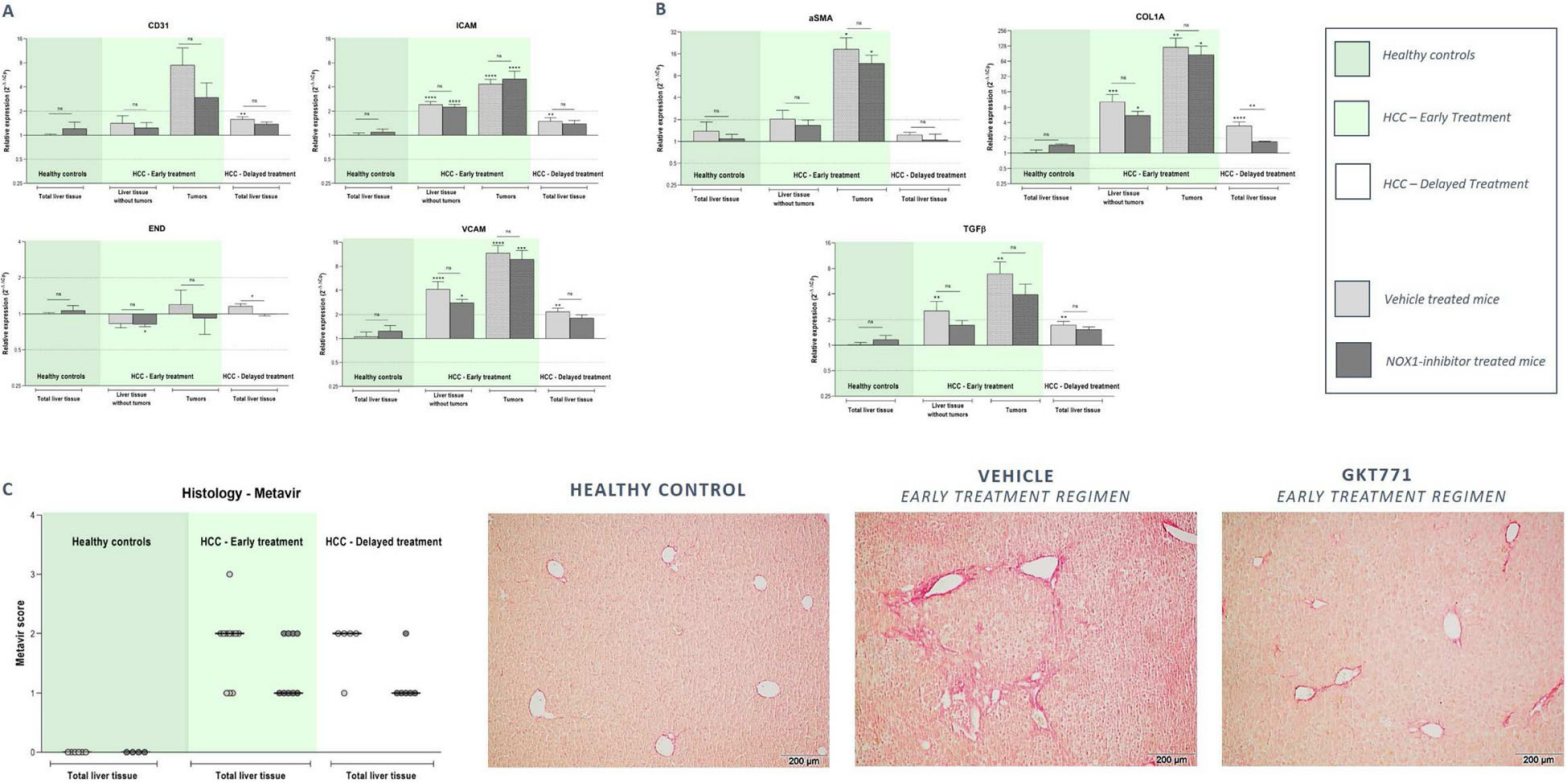
NOX1 inhibition attenuates the induction of an angiogenic and pro-fibrotic microenvironment in advanced experimental HCC. After weekly DEN (35 mg/kg) injections for 25/20 weeks and twice or single daily administration of vehicle/GKT771, healthy, HCC and non-cancerous surrounding liver tissue was sampled for mRNA expression analysis. RT-qPCR was performed to determine the expression of (**a**) different angiogenic markers (CD31, END, iCAM and vCAM) and **b** fibrotic markers (αSMA, COL1A and TGFβ). Data shown as mean ± SEM. ^ns^not significant; * *p* < 0,05; ** *p* < 0,01; *** *p* < 0,001; **** *p* < 0,0001 compared to vehicle-treated healthy controls, unless indicated differently. **c** Livers were stained with Sirius red and Metavir scoring was determined to assess the extent of fibrosis of the histological liver sections. Data shown as individual scoring points with median. Metavir score: F0 - no fibrosis; F1 - fibrosis exists with expansion of portal zones; F2 - fibrosis exists with expansion of most portal zones, and occasional bridging

To evaluate the therapeutic potential of NOX1 inhibition in advanced HCC, two treatment regimens were set-up including an early and delayed treatment protocol (Fig. 1b). In both treatment regimens, DEN administration induced macroscopically visible tumoral lesions in the liver, while in the saline-treated control group no lesions were detected (Fig. 7a-b). Early treatment and twice daily dosing of GKT771 during advanced HCC resulted in reduced HCC lesions compared to no treatment (Fig. 7a-b). This was confirmed by less induced expression of the HCC markers AFP and GP3, both in the early (twice daily dosing) and delayed (single daily dosing) treatment group compared to untreated HCC mice (Fig. 7c). Assessment of the TME of advanced HCC liver tissue showed induction of markers involved in inflammation (IL1β, TNFα, IL6, CCL2, CCR2), inflammasome activation (Caspase 1, NLRP3, iNOS), immune suppression (PDL1), angiogenesis (END, CD31, iCAM, vCAM) and fibrosis (αSMA, TGFβ, COL1A) compared to healthy control livers (Figs. 11, 12 and 13A, light grey bars). In early twice daily dosed treated HCC mice, a (partial) loss of significant induction of IL1β, TNFα, CCL2, CCR2, Caspase 1 and NLRP3 in the HCC lesions could be observed (Fig. 8a). This was also confirmed by multiplex analysis showing reduced CCL2, TNF and IL6 production in NOX1i treated mice. Furthermore, treatment with NOX1 inhibition resulted in downstream inhibited ROS production in both treatment regimen (Fig. 8b). Expression of angiogenic marker vCAM was less induced in both the non-cancerous surrounding liver tissue and the HCC lesions of early twice daily treated HCC mice (Fig. 9a). Fibrosis was attenuated in both early and delayed GKT771 treated HCC mice, indicated by a (partial) loss of significant induction of COL1A and TGFβ expression in the surrounding liver, HCC lesions and total liver tissue (Fig. 9b). Furthermore, both treatment regimens resulted in a reduced Metavir score compared to untreated HCC mice (Fig. 9c).

## Discussion

HCC related morbidity and mortality continues to rise as approximately forty percent of HCC patients have advanced underlying liver disease and are not eligible for potentially curative treatment options. The poor prognosis of advanced HCC is partly due to the limited efficacy and many adverse events of existing systemic treatments [1]. Consequently, there is an absolute medical need for the development and evaluation of new targeted therapies, which could also potentiate the clinical benefits of current systemic therapies. Our current study evaluated the effect of chemical NOX1 inhibition on the development of HCC and its potential as therapeutic strategy with focus on interference with the TME.

The development and progression of HCC occurs in the context of a complex microenvironment associated with many types of cellular stress. These stress pathways and their molecular mediators could serve as adequate candidate targets for such adjuvant systemic treatment strategies. In particular, due to the massive oxygen consumption in the growing tumor, hypoxia is a prevalent feature of the hepatic TME. In a hypoxic environment, persistent ROS accumulation induces inflammatory responses, resulting in genetic instability, chromosomal damage, and tumor development and metastasis. These increased amounts of ROS are generated by non-mitochondrial sources such as NADPH oxidase (NOX) enzymes [29]. High NOX1 levels have been shown to correlate with poor prognosis of HCC patients [24, 25]. Interestingly, in an experimental colon carcinoma model, treatment with a NOX1 inhibitor (GKT771) in combination with an anti-PD1 immune checkpoint inhibitor had an additional inhibitory effect on tumor growth, which suggest a possible therapeutic capacity of combining NOX1 inhibitors in other solid tumors such as HCC [31]. However, studies on this potential benefit of NOX1 inhibition in the prevention or treatment of HCC are still limited.

We first assessed the potential direct cytotoxic effects of NOX1 inhibition on human HCC cells by subjecting Huh7 and Hep3B cells to various concentrations of GKT771. Our results suggest a reduced cellular activity of these human HCC cell lines without cytotoxicity. However, this was not the case in the murine Hepa1-6 cell line, were no concentration-dependent reduction of cellular activity could be detected and cytotoxicity was induced at the highest GKT771 concentration. These data suggest a different sensitivity of human and murine HCC cell lines in *in vitro* NOX1 inhibition.

We next wanted to investigate the anti-tumoral effect of NOX1 inhibition in an *in vivo* setting of orthotopic HCC in the context of a complex microenvironment. Therefore, we used a DEN-induced HCC mouse model to investigate whether chemical NOX1 inhibition is able to reduce HCC development. While we did not observe a reduction in HCC lesions in this preventive setting, NOX1i reduced the induction of tumoral stemness markers indicating the involvement of NOX1 in tumor aggressiveness. Liu et al. [29] previously stated that tumor hypoxia accelerates the aggressiveness of HCC cells due to the close relationship between ROS on the one hand, and EMT, tumor progression, invasiveness, angiogenesis, phenotype conversion and distant metastasis on the other hand. The insufficient oxygen amount in a hypoxic microenvironment limits tumor cell division, facilitates transition of malignant cells towards a more metastatic phenotype and endorses treatment resistance, in which cancer stem cells have been proven to play an important role. Additionally, high expression of NOX1 has been associated with HCC progression and aggressiveness [30].

In order to be able to translate results to a more clinical setting, two treatment regimens of NOX1i were evaluated in advanced/established HCC. Here, early treatment with a double daily dose of NOX1i resulted in an attenuation of HCC progression, which confirms the results published by Liang et al. showing that NOX1-/- knockout mice or WT mice given DEN followed by the NOX-1 inhibitor ML171 developed fewer and smaller tumors after DEN injection. Additional to the investigation of cancerous traits of HCC, we focused on the effects of GKT771 on the tumoral microenvironment during HCC development. Interestingly, pro-inflammatory and angiogenic markers were less induced and fibrosis attenuated upon NOX1 inhibition during HCC progression in mice. Both our preventive and therapeutic studies confirm the ability of NOX1 inhibition to reduce the induction of a pro-inflammatory, angiogenic and pro-fibrotic microenvironment. For the DEN-induced HCC mouse model, it has been shown that NOX1 knockout mice exhibit equal amounts of hepatic DNA damage compared to wild type mice. This suggests that the carcinogenic effect of DEN is not only induced by DNA damage but in addition, ROS use a different mechanism of action generating hepatocyte injury, cell death, inflammation, and compensatory proliferation and fibrosis in the pathogenensis of DEN-induced HCC [26]. As both preventive and therapeutic GKT771 treatment in DEN-induced HCC attenuate the development of a pro-tumorigenic, and especially pro-fibrotic, microenvironment, NOX1 inhibition potentially possesses an adjuvant value to currently tested and available HCC therapies. The TME composes a biological barrier around solid cancers that prevents immunotherapy from effectively working. The TME is acidic, hypoxic and full of toxins and acts like a barricade that is able to fend off or chemically assault cancer-killing immune cells [31]. Furthermore, it has been shown that the TME mediates the resistance of HCC towards tyrosine kinase inhibitors such as Sorafenib, via the regulation of cell stemness, mesenchymal state of tumor cells and epigenetic mechanisms [32].

Different hepatic cell types, including hepatocytes, HSCs and KCs, express NOX1 [8, 22, 33]. However, Liang et al. recently published that NOX1 expression in macrophages, but not in other hepatic cell types, mediates their tumor-promoting activity [26]. We observed that primary murine BMDMs treated with GKT771 displayed a significant enhanced mitochondrial metabolic state, associated with plasma membrane disruption, both in the presence and absence of LPS, suggesting a moderate direct toxic effect of NOX1 inhibition in these immune cells. Therefore, we further assessed the effect of NOX1 inhibition on the functional phenotype of LPS-stimulated BMDMs and the human THP1 monocyte cell line. Remarkably, the LPS-induced increase in expression of inflammatory markers was attenuated during NOX1 inhibition by GKT771 treatment. Our *in vitro* results indeed suggest that direct chemical NOX1 inhibition does not primarily targets hepatic tumor cells, as only a slight reduction in metabolic activity in human HCC cells and only cytotoxic effects at the highest concentration in murine HCC cells could be observed. This might indicate that our observed effects *in vivo* might also be mediated by influencing the polarization of cells characterizing the TME, as also published by Liang et al..

Our results of overall safety of NOX1 inhibition on human HCC cells, the effect of NOX1 inhibition on pro-inflammatory macrophage polarization and the attenuated development of a pro-tumorigenic environment by NOX1 inhibition in experimental HCC, suggests this might be a very promising adjuvant therapeutic strategy in the treatment of advanced HCC.

## Data Availability

The data during and/or analysed during the current study available from the corresponding author on reasonable request.

## Abbreviations

BMDM: Bone-marrow derived macrophages
DEN: Diethylnitrosamine
DMSO: Dimethylsulfoxide
EMT: Epithelial-mesenchymal transition
HCC: Hepatocellular carcinoma
HSCs: Hepatic stellate cells
IP: Intraperitoneal
KCs: Kupffer cells
LDH: Lactate dehydrogenase
LPS: lipopolysaccharide
MTT: 3-(4,5-dimethylthiazol-2- yl)-2,5-difenyltetrazolium bromide
NOX: NADPH oxidase
NOX1i: NOX1 inhibition
ROS: Reactive oxygen species
RT-qPCR: Reverse transcriptase quantitative real-time polymerase chain reaction
TME: Tumor microenvironment
WT: Wild type
